# Repetitive transcranial magnetic stimulation suppresses glia-associated neuroinflammation and promotes peripheral nerve recovery in neuropathic pain

**DOI:** 10.3389/fimmu.2026.1830638

**Published:** 2026-05-07

**Authors:** Daniel Youngsuk Kim, Hye Ryeong Sim, Joo-Wan Choi, Jee In Choi, Xin Yi Yeo, Sungchan Ha, A Reum Je, Sangmi Jun, Jong Moon Kim, Sangyong Jung, MinYoung Kim

**Affiliations:** 1 Research Competency Milestones Program (RECOMP), School of Medicine, CHA University, Seongnam, Republic of Korea; 2 Department of Medicine, School of Medicine, CHA University, Seongnam, Republic of Korea; 3 Department of Rehabilitation Medicine, School of Medicine, CHA Bundang Medical Center, CHA University, Seongnam, Republic of Korea; 4 Department of Medical Science, School of Medicine, CHA University, Seongnam, Republic of Korea; 5 Rehabilitation and Regeneration Research Center, CHA University School of Medicine, Seongnam, Republic of Korea; 6 Department of Psychological Medicine, Yong Loo Lin School of Medicine, National University of Singapore, Singapore, Singapore; 7 Center for Research Equipment, Korea Basic Science Institute, Cheongju, Republic of Korea

**Keywords:** inflammation, microglia, muscle atrophy, myelin sheath, nerve regeneration, neuropathic pain, repetitive transcranial magnetic stimulation, Schwann cell

## Abstract

**Background:**

Neuropathic pain (NP) is a chronic condition caused by peripheral nerve damage and is characterized by persistent neuroinflammation and limited treatment options. Repetitive transcranial magnetic stimulation (rTMS) has been reported to modulate neuroinflammation in the brain. However, it remains unclear whether rTMS also influences inflammatory responses in the spinal cord and peripheral nerve structures.

**Methods:**

A rat NP model was established by unilateral sciatic nerve ligation, and the effects of rTMS were evaluated through behavioral testing and molecular, histological, and ultrastructural analyses of the spinal cord and sciatic nerve.

**Results:**

NP induced thermal hyperalgesia and mechanical allodynia, whereas rTMS significantly alleviated these pain-related behaviors (p < 0.05). In the spinal cord, NP increased the expression of pro-inflammatory markers including CD40, CD86, ionized calcium-binding adapter molecule-1 (Iba-1), and transient receptor potential cation channel subfamily V member 1 (TRPV1) (p < 0.05 for TRPV1; p < 0.01 for the others). rTMS significantly attenuated the increases in CD86, Iba-1, and TRPV1 (p < 0.01 for Iba-1; p < 0.05 for the others), while CD40 showed a decreasing trend without statistical significance. In the sciatic nerve, NP also elevated glial and inflammatory markers (Iba-1, TRPV1, S100, and glial fibrillary acidic protein (GFAP), which were significantly reduced following rTMS treatment (p < 0.01 for S100; p < 0.05 for the others). Immunostaining confirmed a reduction in both the number and activation state of Iba-1(+) and GFAP(+) cells in the rTMS-treated group. Ultrastructural analysis demonstrated improved myelin integrity in the sciatic nerve after rTMS, including increased myelin thickness, higher myelinated axon density, and a reduced G-ratio. rTMS also mitigated NP-induced gastrocnemius muscle atrophy, as indicated by increased muscle mass and cross-sectional area (p < 0.01). rTMS was associated with changes in ERK and Akt signaling pathways that were reduced under NP conditions.

**Conclusion:**

rTMS alleviates NP by suppressing glia-associated neuroinflammation in both the spinal cord and sciatic nerve and by promoting structural recovery of peripheral nerves. These findings support rTMS as a promising non-invasive therapeutic strategy for NP.

## 1 Introduction

Neuropathic pain (NP) is a chronic pain condition caused by a lesion or disease of the somatosensory system, leading to spontaneous pain and hypersensitivity to normally innocuous stimuli (1), affecting 6–8% of the general population (2). However, in 50% of NP patients, significant pain relief is not achieved with standard treatments, which include pharmacotherapy, physiotherapy, and psychotherapy (3). Medications commonly used to manage NP often have considerable side effects, may lead to treatment resistance and abuse, and can exacerbate pain over time (4). Consequently, there is growing interest in non-invasive neuromodulation techniques such as repetitive transcranial magnetic stimulation (rTMS), which shows promise due to its ability to influence neuroplasticity and modify pain perception circuits (5, 6).

The pathogenesis of NP is strongly associated with neuroinflammatory responses at sites of nerve injury, including the activation of microglia, astrocytes, and Schwann cells, as well as increased production of pro-inflammatory cytokines (7–11). Studies have demonstrated that rTMS exerts therapeutic effects by reducing the expression of pro-inflammatory cytokines, including interleukin (IL)-1β, IL-6, and tumor necrosis factor (TNF)-α, while increasing IL-10, a key anti-inflammatory cytokine, in cortical and subcortical brain regions of NP rat models (12–14). Regarding the mechanisms underlying rTMS-induced pain reduction, previous studies have focused primarily on the modulation of neuronal activity in the cortex, thalamus, and injury site to elucidate the beneficial effects of rTMS on pain tolerance (15–18), potentially providing incomplete information regarding somatic pain conduction. Previous studies have suggested the possible involvement of the spinal cord, which relays nociceptive signals, indicating that (19) investigation of this region may provide a more comprehensive understanding of rTMS efficacy. Furthermore, based on recent reports highlighting the role of Schwann cells in NP development and progression (11), this study also incorporated an analysis of their contributions. After peripheral nerve injury, Schwann cells undergo significant changes, including dedifferentiation, proliferation, and release of glial mediators that influence both neuroinflammatory responses and myelin maintenance (20, 21). To date, the direct effect of rTMS on Schwann cells and myelin maintenance in NP remains unexplored. Although previous studies have shown that rTMS can modulate inflammatory responses in NP models (12–14), its effects on glia-associated neuroinflammatory responses and myelin-related structural changes in the spinal cord and injured peripheral nerve remain incompletely understood. Additionally, signaling pathways such as extracellular signal-regulated kinase (ERK) and protein kinase B (Akt), which transmit survival signals regulating cell survival and proliferation in response to growth factors (22, 23), are key mediators of neuroinflammation (24, 25) and myelin regeneration (26, 27).

Therefore, this study aimed to investigate the effects of rTMS in a rat model of NP induced by unilateral sciatic nerve ligation. In particular, we examined whether rTMS modulates glial activation and neuroinflammation in the spinal cord and sciatic nerve and whether these changes are associated with structural and functional recovery. Mechanical and thermal nociceptive behaviors were assessed, and glial responses, including inflammatory changes in the spinal cord tissue and sciatic nerve, were quantified using transient receptor potential cation channel subfamily V member 1 (TRPV1), a key nociceptive marker, to assess rTMS effects. Additionally, the impact of rTMS on NP-induced myelin sheath degradation in the sciatic nerve and muscular atrophy was evaluated. To investigate signaling pathways potentially associated with rTMS-related myelin structural recovery in NP, the ERK and Akt signaling pathways were quantified. These findings may enhance our understanding of the multifaceted therapeutic potential of rTMS in NP by modulating inflammation and promoting neuronal regeneration.

## 2 Materials and methods

### 2.1 Animal husbandry

All animal experiments were approved by the Institutional Animal Care and Use Committee of CHA University (IACUC210143) and conducted in accordance with the guidelines of the Laboratory Animal Research Center, CHA University – Bundang Medical Center. Adult male Sprague–Dawley rats (140–160 g) were purchased from SAMTAKO Ltd. (Osan-si, Gyeonggi-do, Republic of Korea) and acclimatized for seven days in the CHA University Laboratory Animal Research Center before use. The rats were housed in pairs in a temperature- and humidity-controlled facility under a 12-h light/dark cycle (lights on at 7:00 AM).

### 2.2 Establishment of the NP model

**Figure 1:**
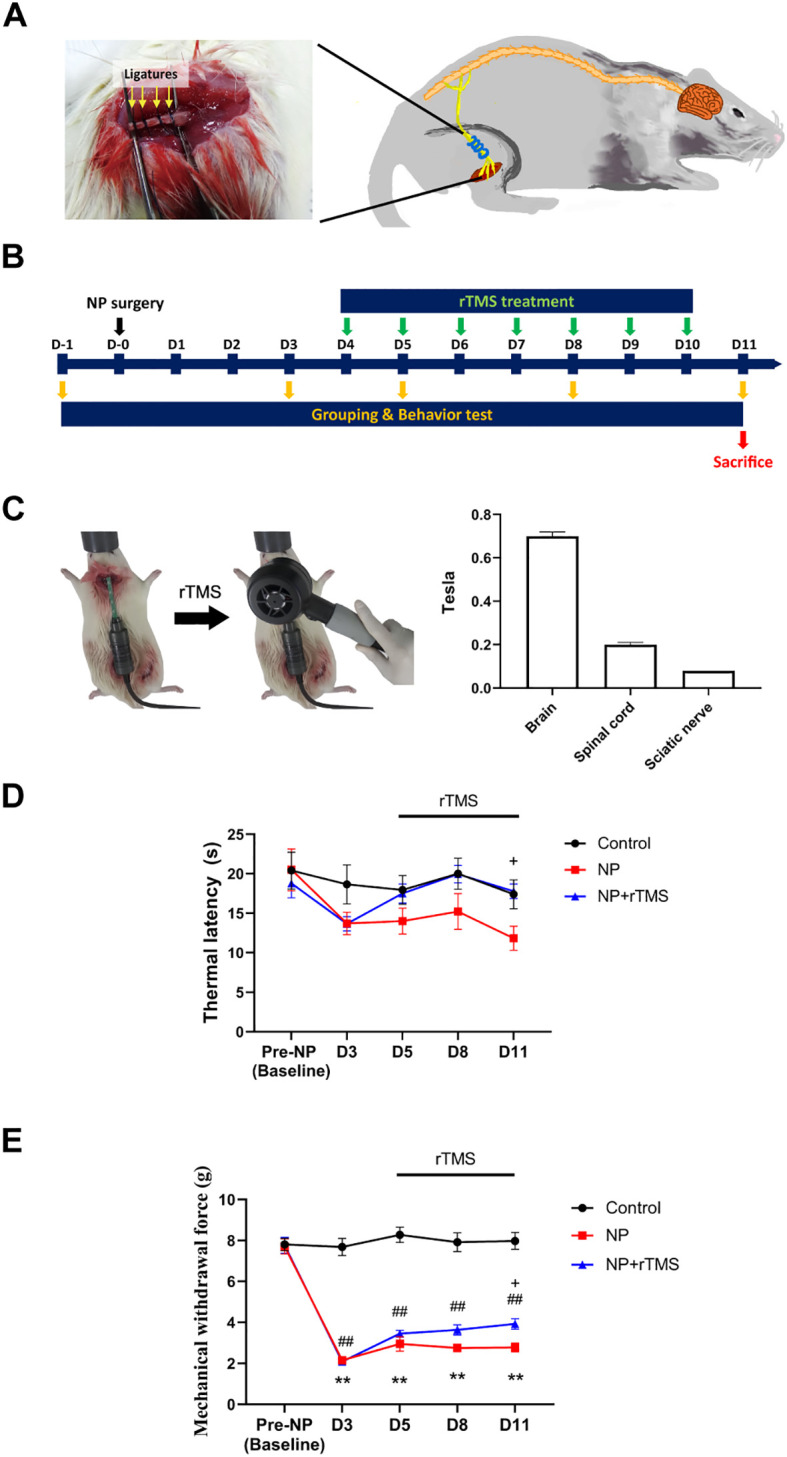
rTMS reverses the thermal hyperalgesia and mechanical allodynia induced by NP induction. **(A)** Schematic illustrating the procedure for sciatic nerve ligation. **(B)** Overall experimental design. NP was induced with unilateral ligation of the right sciatic nerve. Starting on day 4 after surgery, the rTMS group received the treatment for seven consecutive days. On day 11 post-NP establishment, the rats were euthanized. **(C)** A Gaussmeter was inserted into the rat’s brain, spinal cord, and sciatic nerve to measure the intensity of magnetic fields generated by rTMS at each point. The effect of rTMS on the thermal **(D)** and mechanical **(E)** sensory latency in the NP model measured using the hot plate test and the von Frey test, respectively. Two-way repeated-measures ANOVA followed by Tukey’s *post hoc* multiple-comparisons test was used for statistical analysis. Data are presented as mean ± SEM from Control (n = 10), NP (n = 9), and NP+rTMS (n = 9) rats. *p < 0.05 and **p < 0.01 between control and the NP. ^##^ p < 0.01 between control and NP+rTMS. ^+^ p < 0.05 between the NP and NP+rTMS (**Supplementary Tables 2**, **3**).

Prior to NP surgery, rats were randomly assigned to three groups (n = 10 per group) based on baseline behavioral assessments (von Frey and hot plate tests) to ensure no significant preoperative differences among groups. NP was induced in two-thirds of the rats by partial ligation of the right sciatic nerve, following a previously established method (28). Rats were anesthetized with isoflurane, and the right thigh area was shaved. A ~1 cm incision was made on the shaved skin to expose the underlying muscle tissue. The muscle was bluntly dissected to expose the sciatic nerve near the greater trochanter region. The sciatic nerve, which arises from the ventral rami of the fourth lumbar to third sacral spinal nerves, was carefully isolated and partially ligated using Blue Nylon (AILEE, 4.0) (29). The incision was then closed with Black Silk (AILEE, 4.0) sutures (**Figure 1A**). After NP surgery, the rats were assigned to either the NP group (no rTMS treatment) or the NP+rTMS group (rTMS treatment), ensuring comparable baseline behavioral responses before initiation of rTMS. Control group rats did not undergo surgery. Based on prior studies reporting minimal differences between sham-operated and naïve control animals in comparable behavioral and inflammatory outcomes (14, 18), a sham-operated group was not included in the present study. Nevertheless, the potential contribution of surgical exposure and tissue manipulation cannot be fully excluded and should be considered when interpreting the findings.

### 2.3 rTMS treatment protocol

The NP+rTMS group underwent daily rTMS sessions for seven consecutive days at the same time each day, starting day 4 after sciatic nerve ligation (**Figure 1B**). After anesthesia with isoflurane, rTMS was delivered using a round coil that was specifically designed for rat brain stimulation (inner diameter: 5 cm; outer diameter: 7 cm; REMED Ltd., Korea) (19, 30). Using the bregma region as an anatomical reference (31), the center of the round coil was positioned over this point, placed in direct contact with the scalp, and maintained in a fixed orientation parallel to the skull surface during all treatment sessions to ensure consistent positioning across animals and sessions. Stimulation consisted of 40 trains of 20 Hz pulses delivered for 2 seconds per train, with a 28-second inter-train interval, resulting in 1,600 pulses per session (30). High-frequency stimulation (20 Hz) was selected based on previous studies demonstrating analgesic and anti-inflammatory effects of rTMS (30, 32). The stimulation intensity at the coil surface was set at 0.7 T, and magnetic field intensities at the spinal cord and sciatic nerve were measured separately using a Gaussmeter (TM-801EXP, KANETEC CO. Ltd., Japan) (**Figure 1C**). During rTMS administration, no seizures, abnormal behaviors, or signs of distress were observed, although occasional scalp or facial muscle twitching occurred during stimulation.

### 2.4 Pain behavioral tests

#### 2.4.1 Von Frey test

Mechanical hyperalgesia was assessed using an automatic von Frey dynamic plantar aesthesiometer (DPA; Ugo Basile, Italy). Rats were acclimatized in plexiglass cages with a metal wire grid floor for 10 min before testing. A 0.5 mm filament was applied to the plantar surface of the right hind paw, with force increasing gradually from 0 to 10 g at a rate of 1 g/s. The mechanical stimulus was terminated upon paw withdrawal. Behavioral measurements were performed before NP induction to establish baseline sensitivity and to ensure comparable distribution of the rats across the experimental groups. On day 3 after surgery, behavioral tests were repeated to confirm NP induction, after which NP animals were assigned to either the NP group or the NP+rTMS group. Repeated measurements were performed on days 5, 8, and 11 during or after rTMS treatment. Each measurement consisted of three consecutive trials with an interval of at least 30 seconds between trials (33, 34). All behavioral assessments were performed at consistent time points by an investigator blinded to group allocation.

#### 2.4.2 Hot plate test

A Hot/Cold Plate apparatus (Ugo Basile, Italy) was used to evaluate thermal hyperalgesia in rats. Each rat was placed on a stainless-steel plate maintained at 50 ± 0.1 °C. The latency from placement on the plate to the first observed nociceptive response, such as foot licking or rapid paw withdrawal, was recorded. To prevent tissue damage, a cutoff time of 60 seconds was set, after which the rat was removed from the plate. The test was conducted before NP surgery and on days 3, 5, 8, and 11 post-surgery (34, 35). All measurements were performed by an investigator blinded to group allocation.

### 2.5 Quantitative real-time polymerase chain reaction

Total RNA was extracted from rat spinal cord tissue using a phenol/chloroform method with TRIzol reagent (#15596018, Invitrogen, USA), following the manufacturer’s protocol. RNA concentration was determined using a NanoDrop 2000 Spectrophotometer (Thermo Fisher Scientific, USA) by measuring optical density at 260 nm. First-strand complementary DNA was synthesized using the Maxime RT PreMix Kit (#25081, iNtRON, USA) with 1 μg of total RNA and the provided oligo primer, following the manufacturer’s instructions. qRT-PCR was performed using the CFX Connect Real-Time PCR Detection System (#1855201, Bio-Rad, USA), with AccuPower 2X GreenStar qPCR Master Mix (#K-6254, Bioneer, USA) and 100 ng of complementary DNA per reaction. All reactions were performed in duplicate, with a no-template control included to ensure specificity. Gene expression levels were analyzed using the 2^–ΔΔCt^ method (36) and expressed as ratios relative to the internal control, glyceraldehyde-3-phosphate dehydrogenase. The primers used for qPCR analysis are listed in **Supplementary Table 1**.

### 2.6 Western blotting

Proteins were extracted from the spinal cord and sciatic nerve using RIPA lysis and extraction buffer (#89901, Thermo Fisher Scientific), supplemented with protease inhibitors (#P8340, Sigma-Aldrich, USA) and phosphatase inhibitors (#P5726, Sigma-Aldrich). Tissue samples were physically ground using a sonicator (S-450D, Branson, USA) before centrifugation at 15,000 × g for 20 minutes at 4 °C. The supernatant was collected, and protein concentration was determined using the Pierce BCA Protein Assay (#23227, Thermo Fisher Scientific). Samples were prepared with Laemmli sample buffer (S3401, Sigma-Aldrich) and heated at 95 °C for 5 minutes before gel electrophoresis. A total of 20 μg of protein was separated on 10% or 12% sodium dodecyl sulfate-polyacrylamide gels and transferred onto 0.45 μm polyvinylidene fluoride membranes (IPVH00010, Millipore, USA) using a wet transfer system (25 mM Tris, 192 mM glycine, and 20% methanol). Membranes were blocked with 5% skim milk (232100, BD Difco, USA) in Tris-buffered saline containing 0.1% Tween-20 for 1 hour at room temperature, followed by overnight incubation at 4 °C with primary antibodies: rabbit polyclonal anti-TRPV1 (#NB100-1617, Novus Biologicals, USA), mouse monoclonal anti-ERK1/2 (#SC-514302, Santa Cruz Biotechnology, USA), rabbit monoclonal anti-p-ERK1/2 (Thr202/Tyr204; #4370, Cell Signaling Technology, USA), rabbit monoclonal anti-Akt (#4691, Cell Signaling Technology), rabbit monoclonal anti-p-Akt (Ser473; #4060, Cell Signaling Technology), mouse monoclonal anti-β-actin (#SC-47778, Santa Cruz Biotechnology), rabbit polyclonal anti-ionized calcium-binding adapter molecule-1 (Iba-1) (#019-19741, Fujifilm Wako, Osaka, Japan), mouse monoclonal anti-S100 (#5529, Cell Signaling Technology), and rabbit polyclonal anti-glial fibrillary acidic protein (GFAP) (#ab7260, Abcam, UdK). Membranes were then incubated with horseradish peroxidase (HRP)-conjugated secondary antibodies: anti-mouse IgG (#7076, Cell Signaling Technology) and anti-rabbit IgG (#7074, Cell Signaling Technology) at 1:10,000 dilution in blocking solution for at least 1 hour at room temperature. Protein bands were visualized using Immobilon Western Chemiluminescent HRP Substrate (#WBKLS0500, Millipore) and detected with a luminescent image analyzer (ImageQuant™ LAS 4000, GE Healthcare, USA).

### 2.7 Immunohistochemistry examination for sciatic nerve

Sciatic nerve tissues were sectioned to 10 µm thickness using a cryotome (Leica, Buffalo Grove, IL, USA) and mounted onto HistoBond® adhesive microscope slides. The sections were washed in phosphate-buffered saline (PBS) and incubated for 1 h in blocking solution (2% normal goat serum, 1% triton X-100) at room temperature. They were then incubated overnight at 4 °C with primary antibodies diluted in blocking solution, including 1:100 anti-GFAP (#3670, Cell Signaling Technology) and 1:1000 anti-ionized calcium-binding adapter molecule-1 (Iba-1) (#019-19741, Fujifilm Wako). Secondary antibodies (Alexa Fluor 594 goat anti-rabbit IgG, A-11012, Invitrogen; Alexa Fluor 488 goat anti-mouse IgG, A-11001, Invitrogen) were applied at 1:1,000 dilution and incubated for 3 h at room temperature. Immunohistochemical analysis was performed using Fiji software, and epifluorescence images were captured under identical conditions using an Eclipse Ts2 microscope (Nikon Instruments, Japan). Total cell count was obtained by quantifying 4′,6-diamidino-2-phenylindole (DAPI)(+) cells, while Iba-1(+) cells were identified by co-localization of DAPI(+) and Iba-1(+) staining. Fluorescence intensity was quantified by subtracting the background signal from the raw fluorescence image, followed by measuring median fluorescence intensity within the positive cell regions. For GFAP analysis, background subtraction was uniformly applied to all GFAP immunofluorescence images to ensure consistency.

### 2.8 Ultrastructure imaging of sciatic nerve

Sciatic nerves were fixed in 2.5% glutaraldehyde in 0.1 M cacodylate buffer (pH 7.3) at room temperature. After fixation, tissues were treated with 2% osmium tetroxide and 3% potassium ferrocyanide in 0.1 M cacodylate buffer (pH 7.3) for 1 h at 4 °C in the dark. The samples were then dehydrated using a graded ethanol and propylene oxide series and embedded in Epon 812 resin. Polymerization was performed with pure resin at 70 °C for 48 h. Ultrathin sections (70 nm thick) were obtained using an ultramicrotome (EM UC7, Leica, Austria) and collected onto 150-mesh copper grids. Sections were stained with Uranyless (5 min) and lead citrate (3 min) before being examined using transmission electron microscopy (TEM) at 120 kV (JEM-1400 Plus, JEOL, Japan) at the Research Equipment Center, Korea Basic Science Institute. The thickness of myelin and G-ratio were quantified using MyelTracer software (37), while the density of myelinated axons was measured using Fiji software (National Institutes of Health, USA). Myelin thickness was calculated as the difference between the outer and inner radius of the myelin sheath. The G-ratio was defined as the ratio of the inner-to-outer diameter of a myelinated axon (38). Myelinated axon density was determined by dividing the total combined area of all myelinated axons by the whole image area.

### 2.9 Gastrocnemius muscle staining and cross-sectional area measurement

On Day 11, following behavioral tests, rats were anesthetized with isoflurane. The heart was perfused with PBS, and the gastrocnemius muscle was isolated and post-fixed in 4% formaldehyde for at least 48 h at room temperature. Fixed tissues were rinsed with water to remove residual paraformaldehyde, dehydrated through a graded ethanol series (70%, 95%, 100%), and cleared with xylene. The tissues were then embedded in paraffin wax, sectioned at 5 μm thickness, and mounted onto HistoBond® adhesive microscope slides (#0810001, Marienfeld-Superior, Germany). Sections were deparaffinized in xylene, rehydrated through an ethanol gradient (100%, 95%, 70%), and rinsed with deionized water before staining with hematoxylin and eosin. After staining, sections were washed with water, dehydrated through 95% and 100% ethanol, and cleared with xylene (two dips) before mounting with Vectashield Antifade Mounting Media (#H-1000-10, Vector Laboratories, Newark, CA, USA). Muscle sections were examined using an Eclipse Ts2 microscope (Nikon Instruments, Japan), and cross-sectional area (CSA) was measured using Fiji software (National Institutes of Health). CSA was determined by manually delineating the boundaries of individual muscle fibers on captured hematoxylin and eosin micrographs. Multiple axons and muscle fibers were measured from each animal. For group comparisons, values from individual axons and fibers were first averaged within each animal, and the resulting animal-level mean values (n = animals per group) were used for statistical testing.

### 2.10 Statistical analysis

All data are presented as mean ± standard error of the mean (SEM). Sample size for behavioral experiments was determined *a priori* by power analysis (α = 0.05, power = 0.90) based on effect sizes derived from previous studies (14, 18). For molecular analyses (qPCR and Western blot), all reported n values represent biological replicates from independent animals, with final sample sizes determined by tissue availability and predefined quality control criteria. Statistical analyses were performed using GraphPad Prism 8.0.1 (GraphPad Software, San Diego, CA, USA). Normality of the datasets was assessed using the Shapiro–Wilk test, and homogeneity of variance was evaluated using the Brown–Forsythe test. For normally distributed data, statistical significance was determined using one-way analysis of variance (ANOVA) followed by Tukey’s multiple-comparison *post hoc* test. For non-normally distributed data, the Kruskal–Wallis test was used, followed by Dunn’s multiple-comparison *post hoc* test. Behavioral data obtained from the von Frey and hot plate tests were analyzed using two-way repeated-measures ANOVA (group × time). Tukey’s multiple-comparison test was used for *post hoc* analyses. All reported n values represent biological replicates from distinct individual animals. A p-value < 0.05 was considered statistically significant.

## 3 Results

### 3.1 rTMS ameliorates thermal hyperalgesia and mechanical allodynia in NP rats

A rat model of NP was established by unilateral sciatic nerve ligation (**Figure 1A**). NP-induced rats exhibited gait disturbances, confirming successful model induction (**Supplementary Figure 1**). In the hot plate test, two-way repeated-measures ANOVA revealed significant main effects of time and treatment group (both p < 0.05), with no significant group × time interaction. In the von Frey test, significant main effects of time and treatment group as well as a significant group × time interaction were observed (all p < 0.01).Following NP induction (D3), both the NP and NP+rTMS groups exhibited significantly lower withdrawal thresholds than the control group (p < 0.01) and their respective baselines, indicating successful establishment of NP (p < 0.01) (**Figure 1E**, **Supplementary Table 3**). rTMS generated magnetic fields at the stimulation site (brain), with progressively lower field strengths measured at the spinal cord and sciatic nerve (**Figure 1C**; Brain: 0.70 ± 0.02 T, Spinal cord: 0.20 ± 0.01 T, Sciatic nerve: 0.08 ± 0.00 T). Nevertheless, the NP+rTMS group exhibited increased thermal latency in the hot plate test compared with the NP group (p < 0.05) (**Figure 1D**, **Supplementary Table 2**) by D11. Similarly, mechanical withdrawal thresholds in the von Frey test were higher in the NP+rTMS group than in the NP group (p < 0.05), indicating partial recovery from mechanical hypersensitivity by D11 (**Figure 1E**, **Supplementary Table 3**).

### 3.2 rTMS reduces pro-inflammatory marker expressions in the spinal cord of NP rats

**Figure 2:**
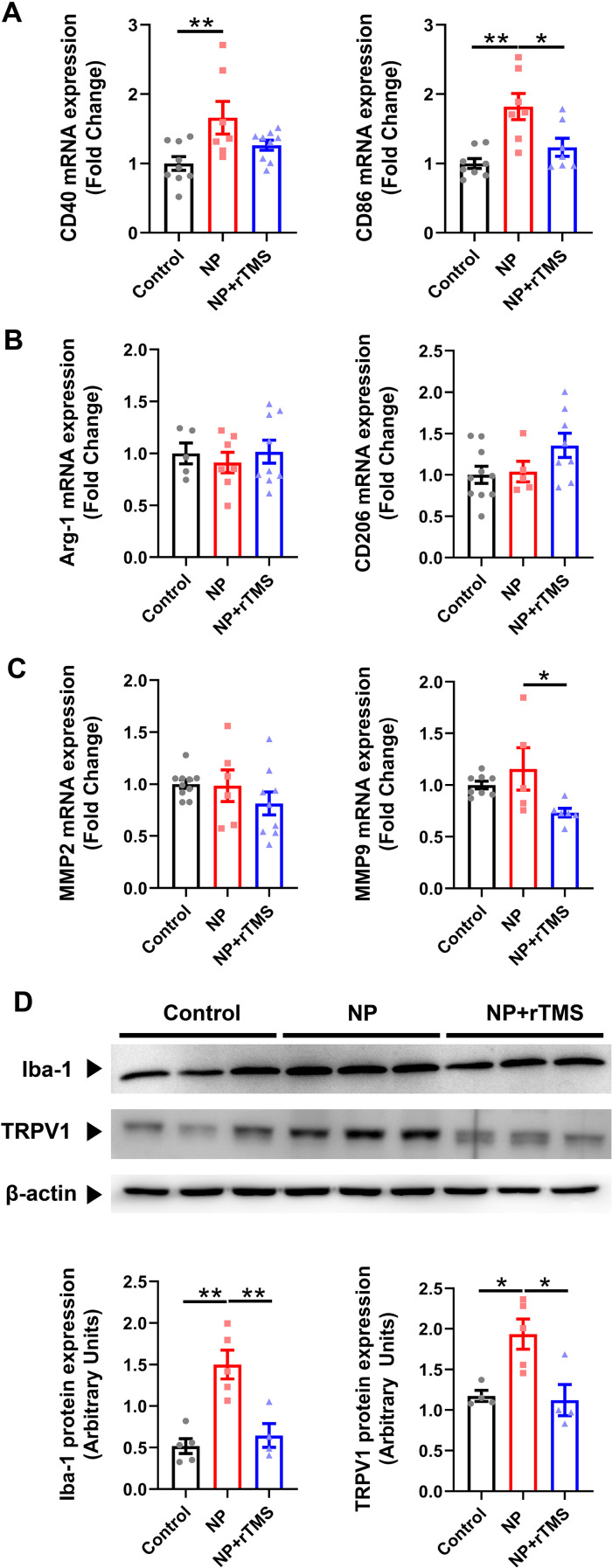
rTMS reduces the expression of proinflammatory markers in the spinal cord of NP rats. Quantification of the mRNA levels of M1 polarization markers, CD40 (**p = 0.0083 Control vs NP group, p = 0.1382 NP vs NP+rTMS group) and CD86 (**p = 0.0009 Control vs NP group, *p = 0.0179 NP vs NP+rTMS group) **(A)**, M2 polarization markers, Arg-1 and CD206 **(B)**, and MMPs, MMP2, and MMP9 (*p = 0.0294 NP vs NP+rTMS group) **(C)** in the spinal cord. **(D)** Western blot quantification of the protein expression of Iba-1(**p = 0.0009 Control vs NP group, **p = 0.0038 NP vs NP+rTMS group) and TRPV1 (*p = 0.02 Control vs NP group, *p = 0.0139 NP vs NP+rTMS group) in the spinal cord of each group. One-way ANOVA or Kruskal–Wallis test was used for the statistical analysis. Data are presented as mean ± SEM (qRT-PCR: n = 5–10 per group; Western blot: n = 4–6 per group).

To evaluate inflammatory responses in the spinal cord following NP induction (39), the expression of inflammatory markers was quantified. qRT-PCR analysis revealed a significant increase in the expression of the M1 polarization markers CD40 and CD86 in the spinal cord tissue of NP rats compared with the control group (both p < 0.01) (**Figure 2A**). CD86 expression was significantly lower in the NP+rTMS group than in the NP group (p < 0.05), whereas CD40 expression showed a decreasing trend that did not reach statistical significance (p = 0.1382) (**Figure 2A**). In contrast, M2 polarization markers, Arginase 1 (Arg-1) and CD206, were not significantly affected by either NP induction or rTMS treatment (**Figure 2B**). Expression of matrix metalloproteinase-9 (MMP9) mRNA was significantly lower in the NP+rTMS group than in the NP group (p < 0.05), whereas MMP2 expression showed no significant differences among groups (**Figure 2C**). Consistent with the qRT-PCR results, Western blot analysis showed that Iba-1 (p < 0.01) and TRPV1 (p < 0.05) protein levels were elevated in the spinal cord of NP rats compared with controls. Both Iba-1 (p < 0.01) and TRPV1 (p < 0.05) protein levels were lower in the NP+rTMS group than in the NP group (**Figure 2D**).

### 3.3 rTMS reduces glial marker expression in the sciatic nerve of NP rats

**Figure 3:**
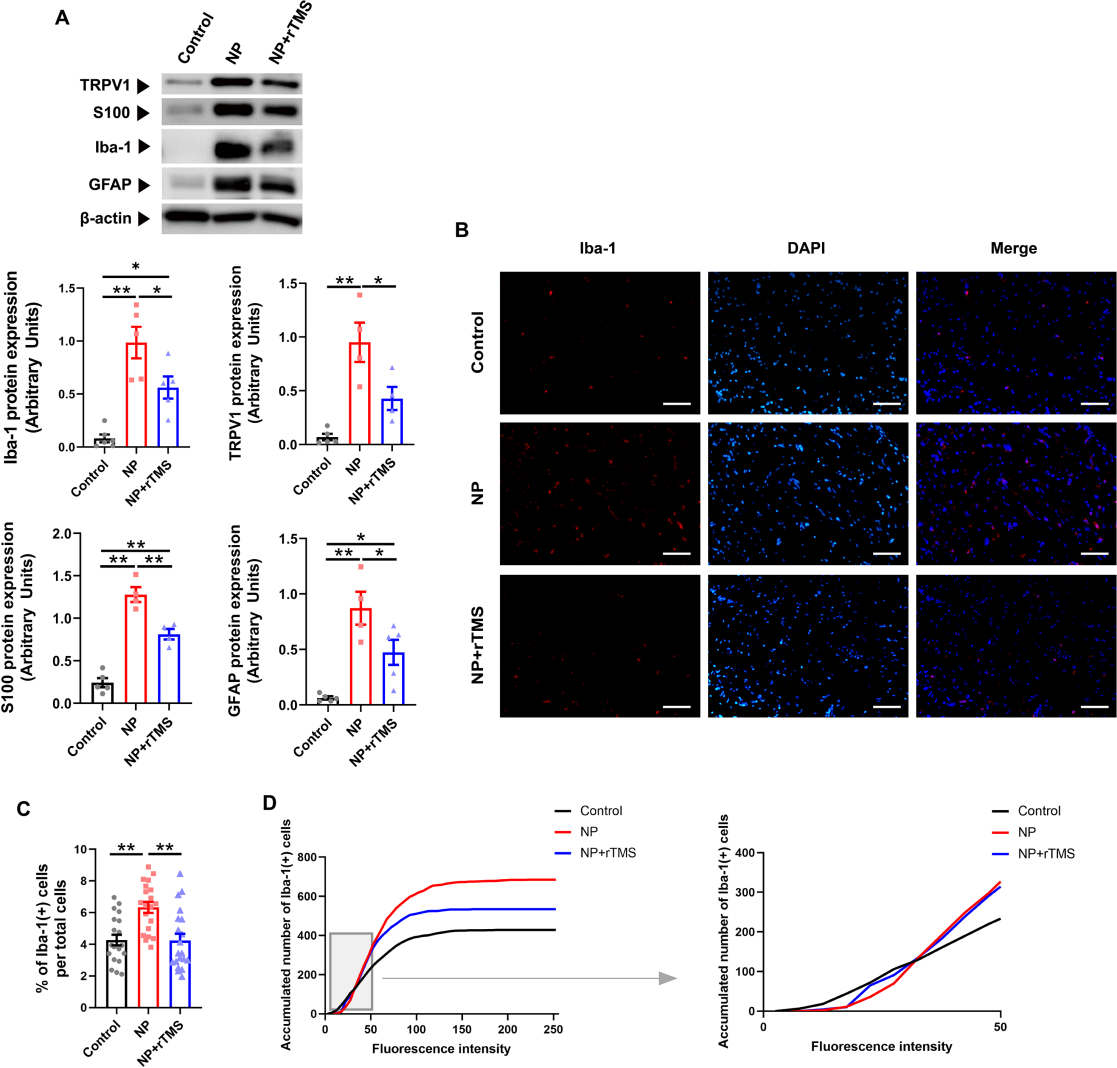
rTMS reduces the expression of glia-associated proteins in the sciatic nerve of NP rats. **(A)** Western blot quantification of the protein expression of Iba-1 (**p < 0.0001 Control vs NP group, *p = 0.012 Control vs NP+rTMS group, *p = 0.0315 NP vs NP+rTMS group), TRPV1 (**p = 0.0006 Control vs NP group, *p = 0.0264 NP vs NP+rTMS group), S100 (**p < 0.0001 Control vs NP group, **p = 0.0003 Control vs NP+rTMS group, **p = 0.0021 NP vs NP+rTMS group), and GFAP (**p = 0.0005 Control vs NP group, *p = 0.031 Control vs NP+rTMS group, *p = 0.0484 NP vs NP+rTMS group) in the sciatic nerves of the Control, NP, and NP+rTMS rats. **(B)** Representative immunofluorescence images for Iba-1 (red), DAPI (blue), and merged in the sciatic nerve of each group. Scale bar: 50 μm. **(C)** Quantitative analysis of the number of Iba-1(+) cells per total cells (**p = 0.0006 Control vs NP group, **p = 0.0005 NP vs NP+rTMS group) and **(D)** accumulated number of Iba-1(+) cells per fluorescence intensity (Left) with a magnified view focusing on low-intensity levels (0–50, right). One-way ANOVA or Kruskal–Wallis test was used for the statistical analysis. Data are presented as mean ± SEM (Western blot: n = 4–6 per group; immunostaining: 20 samples from n = 4 per group).

To assess glial responses in the sciatic nerve following NP induction, protein expression of macrophage and Schwann cell markers was quantified. Protein expression of Iba-1, TRPV1, S100, and GFAP was significantly elevated in the NP group compared with the control group (p < 0.01 for all markers). Expression levels of these markers were lower in the NP+rTMS group than in the NP group (p < 0.01 for S100; p < 0.05 for the others) (**Figure 3A**).

To further examine macrophage activation, immunofluorescence images of Iba-1 was analyzed by quantifying the percentage of Iba-1(+) cells per total cell count and the accumulated number of Iba-1(+) cells per fluorescence intensity. Consistent with the Western blot results, number of Iba-1(+) cells was higher in the NP group than in the control group (p < 0.01) and was lower in the NP+rTMS group than in the NP group (p < 0.01) (**Figures 3B, C**). Fluorescence intensity analysis (40) showed that Iba-1(+) cells in the NP group exhibited higher fluorescence intensity than those in the control group, whereas Iba-1(+) cells in the NP+rTMS group exhibited intermediate fluorescence intensity levels (**Figure 3D**). Similarly, GFAP immunostaining demonstrated increased GFAP(+) cells in the sciatic nerve of NP rats compared with controls, whereas GFAP(+) cell numbers were lower in the NP+rTMS group than in the NP group (**Supplementary Figure 2**).

### 3.4 rTMS improves myelin structure in the ligated sciatic nerve and mitigates gastrocnemius muscle atrophy

**Figure 4:**
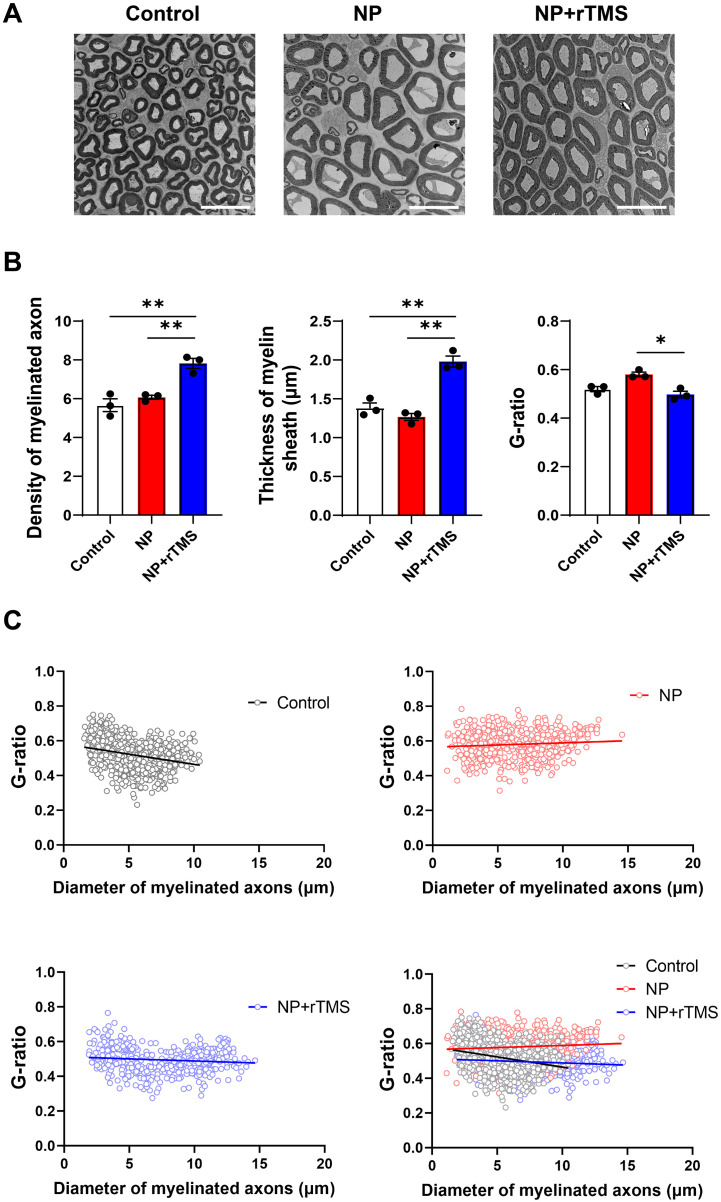
rTMS recovers the morphology of the myelin sheath of the sciatic nerve of NP rats. **(A)** Representative TEM images of the transverse sections of sciatic nerves of the Control, NP, and NP+rTMS rats. Scale bar: 20 μm. **(B)** Quantification of the density of myelinated axons (p = 0.5132 Control vs NP group, **p = 0.0021 Control vs NP+rTMS group, **p = 0.0060 NP vs NP+rTMS group), thickness of myelin sheath (p = 0.4157 Control vs NP group, **p = 0.0011 Control vs NP+rTMS group, **p = 0.0004 NP vs NP+rTMS group), and G-ratio (p = 0.4081 Control vs NP group, p = 0.8902 Control vs NP+rTMS group, *p = 0.0338 NP vs NP+rTMS group) of the sciatic nerves from each group. **(C)** Correlation between the G-ratio and the diameter of myelinated axon in the sciatic nerve of Control, NP, and NP+rTMS rats. One-way ANOVA or Kruskal–Wallis test was used for the statistical analysis. Data are presented as mean ± SEM (All n = 3 per group, Thickness and G-ratio: 479–936 samples, density: 9 samples).

**Figure 5:**
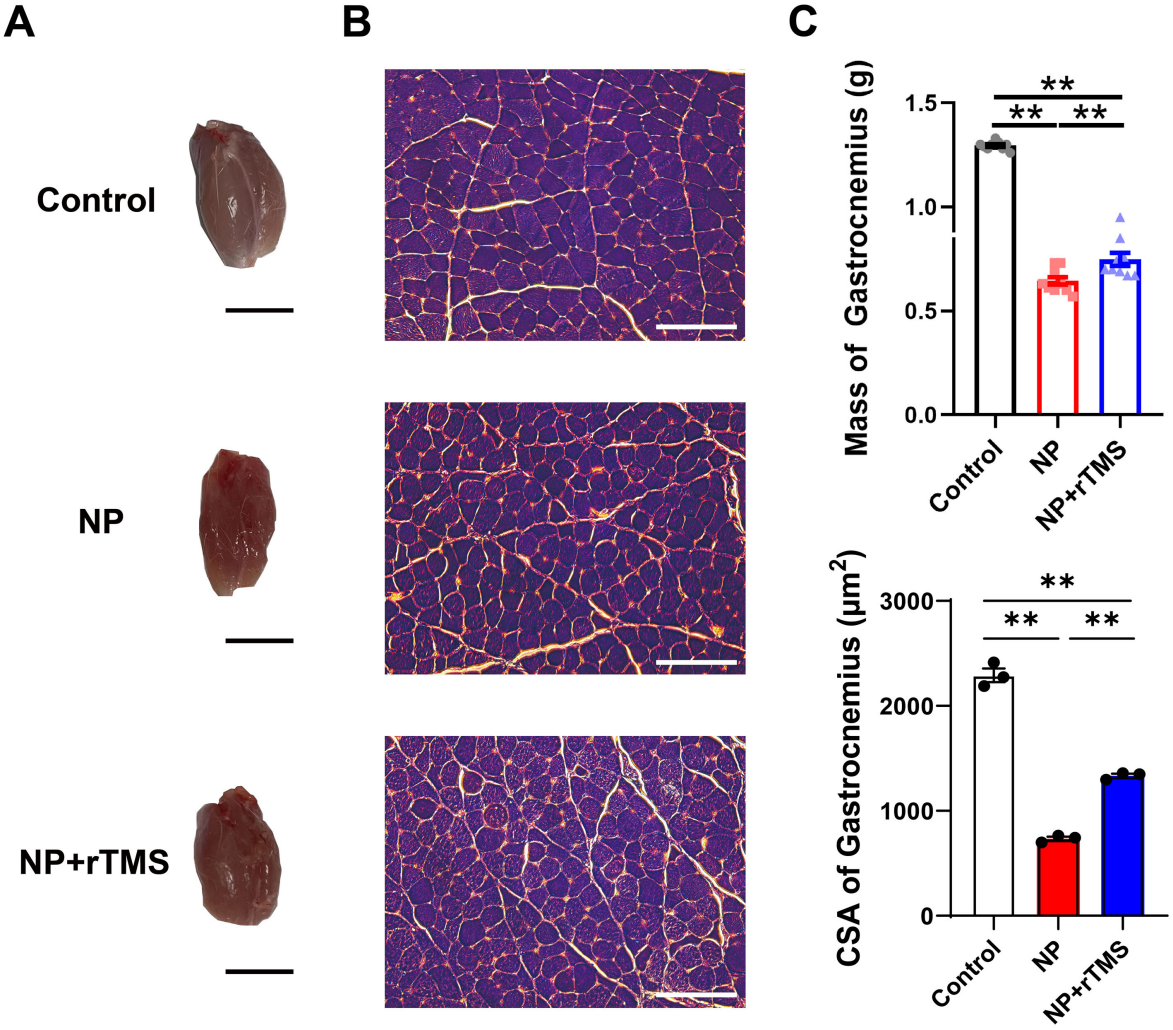
rTMS recovers the morphology of the gastrocnemius muscle of the sciatic nerve of NP rats. Quantification of the effect of rTMS on the morphology, mass, and CSA of gastrocnemius muscle of the NP rats. **(A)** Representative images of gastrocnemius muscles of the Control, NP, and NP+rTMS rats. Scale bar: 10 mm. **(B)** Images of the muscle fiber stained with hematoxylin and eosin. Scale bar: 100 μm. **(C)** Quantification of the mass weight (**p < 0.0001 Control vs NP group, **p < 0.0001 Control vs NP+rTMS group, **p = 0.0064 NP vs NP+rTMS group) and CSA of gastrocnemius muscles (**p < 0.0001 Control vs NP group, **p < 0.0001 Control vs NP+rTMS group, **p = 0.0001 NP vs NP+rTMS group). One-way ANOVA was used for the statistical analysis. Data are presented as mean ± SEM (muscle mass: Control (n = 10), NP (n = 9), NP+rTMS (n = 9); CSA: n = 3 per group, 742–1028 samples).

To evaluate structural changes following NP induction and rTMS treatment, myelin morphology in the sciatic nerve, which is closely associated with Schwann cell function (41), and gastrocnemius muscle parameters, which reflect Wallerian degeneration (42) and neurogenic atrophy (43), were assessed based on animal-level mean values. The density of myelinated axons and myelin sheath thickness were higher in the NP+rTMS group than in the NP group (both p < 0.01), with values exceeding those observed in the control group (both p < 0.01) (**Figure 4**). NP-induced myelin degradation was also reflected by an increased G-ratio in the NP group compared with the control group. The G-ratio was lower in the NP+rTMS group than in the NP group (p < 0.05), whereas compared with the control group, it showed a decreasing trend that did not reach statistical significance (p = 0.8902). Scatter plots further supported this finding, indicating lower G-ratio values across axon diameters in the NP+rTMS group compared with both the NP and control groups (**Figure 4**). NP induction was associated with gastrocnemius muscle atrophy, as both muscle mass and CSA were significantly lower in the NP and NP+rTMS groups than in the control group (both p < 0.01). However, both muscle mass and CSA were higher in the NP+rTMS group than in the NP group (both p < 0.01) (**Figure 5**).

### 3.5 rTMS is associated with changes in ERK and Akt signaling in the spinal cord and sciatic nerve of NP rats

**Figure 6:**
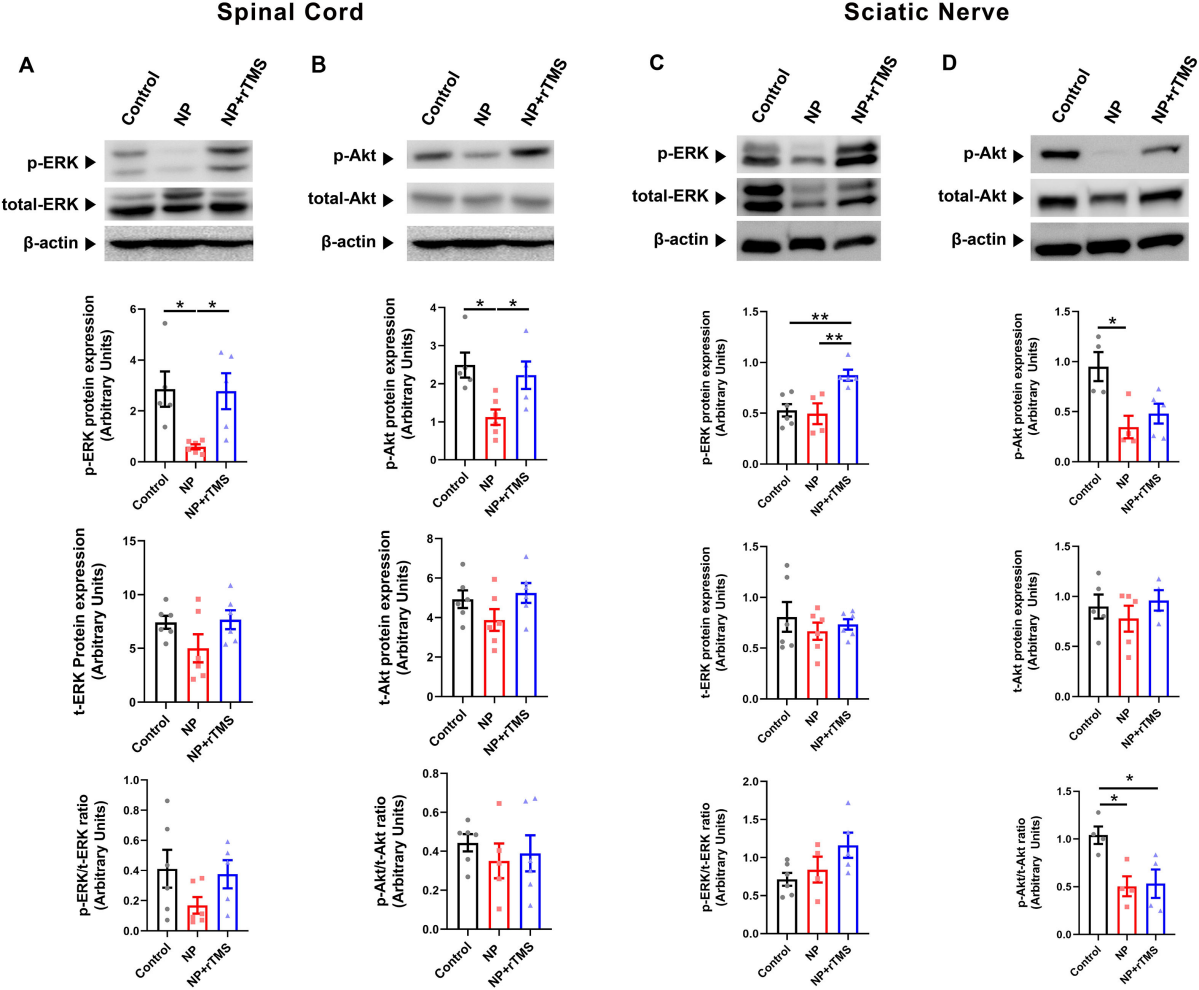
rTMS is associated with changes in ERK and Akt protein levels in the spinal cord and sciatic nerve of NP rats. Western blot quantification of the protein expression of **(A, C)** p-ERK [**(A)** *p = 0.0246 Control vs NP group, *p = 0.0301 NP vs NP+rTMS group, **(C)** **p = 0.0093 Control vs NP+rTMS group, **p = 0.0097 NP vs NP+rTMS group], t-ERK, and p-ERK/t-ERK ratio, and **(B, D)** p-Akt [**(B)** *p = 0.0138 Control vs NP group, *p = 0.0459 NP vs NP+rTMS group, **(D)** *p = 0.0331 Control vs NP group], total Akt, and p-Akt/t-Akt ratio [**(D)** *p = 0.0153 Control vs NP group, *p = 0.0277 Control vs NP+rTMS group)] of the spinal cord and the sciatic nerve, respectively, of the Control, NP, and NP+rTMS rats. One-way ANOVA or Kruskal–Wallis test was used for the statistical analysis. Data are presented as mean ± SEM (n = 4–6 per group].

To evaluate signaling pathways potentially associated with rTMS treatment, ERK and Akt phosphorylation levels were analyzed in the spinal cord and sciatic nerve using Western blot. In the spinal cord, NP induction was associated with lower p-ERK and p-Akt levels compared with the control group (both p < 0.05). Both p-ERK and p-Akt levels were higher in the NP+rTMS group than in the NP group (both p < 0.05) (**Figures 6A, B**). In the sciatic nerve, NP induction was associated with lower p-Akt levels compared with the control group (p < 0.05), whereas p-ERK levels were higher in the NP+rTMS group than in the NP group (p < 0.01) (**Figures 6C, D**). Total ERK and Akt protein levels in the spinal cord showed patterns similar to those of their phosphorylated forms across the groups. Accordingly, the ratios of p-ERK/t-ERK and p-Akt/t-Akt were not significantly different between the NP+rTMS and NP groups. In the sciatic nerve, the p-Akt/t-Akt ratio was lower in the NP group than in the control group (p < 0.05), whereas no significant difference was observed between the NP+rTMS and NP groups (**Figure 6**).

## 4 Discussion

Our previous clinical study demonstrated that high-frequency rTMS (20 Hz, 5-second train duration, 55-second intertrain interval, totaling 2,000 pulses per session over 20 minutes) applied to the ipsilesional dorsolateral prefrontal cortex in stroke patients was effective in enhancing cognition. This therapeutic effect was strongly correlated with an anti-inflammatory response in patients with vascular dementia (44). Notably, variations in inflammatory responses are closely linked to the severity of chronic pain (45). While rTMS has been shown to modulate inflammation (12, 46, 47) and is associated with mild, well-tolerated side effects (48, 49), the precise mechanisms underlying its beneficial effects on NP remain incompletely understood.

The mechanistic pathways underlying the development and perception of persistent pain remain largely unknown, primarily due to the complexity of accurately replicating the multidimensional nature of pain pathophysiology (50). In this study, we utilized a partial sciatic nerve ligation rat model to induce NP, which effectively mimics clinical phenotypes observed in patients with causalgia (51, 52). To evaluate the physiological impact of rTMS on NP, we quantified its effects on mechanical and thermal allodynia, as well as hyperalgesia, in NP rats (**Figures 1D, E**). These behavioral findings indicate that rTMS treatment was associated with attenuation of NP-related hypersensitivity.

The analgesic effects of rTMS are believed to be linked to its ability to modulate neuroinflammation (12, 18). While numerous studies have demonstrated that rTMS reduces neuroinflammation in the cortex, research on its effects in the spinal cord and sciatic nerve remains limited and requires further investigation (13, 14, 53). Notably, in this study, the magnetic field intensity generated by rTMS was primarily concentrated in the brain region, while substantially lower levels were observed in the spinal cord and sciatic nerve (**Figure 1C**). This pattern suggests that the changes observed in distal neural tissues are unlikely to be explained solely by direct magnetic field exposure and may instead reflect indirect downstream modulation through central pain-regulatory pathways.

We observed a targeted reduction in NP-induced upregulation of Iba-1 and TRPV1 protein expression following rTMS treatment, indicating decreased microglial/macrophage and reduced nociceptive transmission in both the spinal cord and sciatic nerve (**Figures 2D**, **3A**). Immunostaining analysis corroborated these findings, demonstrating a reduction in both the number and fluorescence intensity of Iba-1(+) cells in the sciatic nerve of NP rats following rTMS treatment (**Figures 3B–D**). Consistent with our findings, previous studies have reported increased microglial and macrophage activation under NP conditions (54–56). Additionally, minocycline, a known inhibitor of microglial activation, has been shown to effectively alleviate NP, suggesting that rTMS may promote NP recovery through a similar mechanism involving modulation of microglial activity (57–59).

Previous studies have suggested that counteracting NP involves inhibiting M1 microglia and promoting M2 polarization, particularly in the spinal cord (9, 60). In line with this, our study demonstrated a reduction in CD86 expression, an M1 marker, while Arg-1 and CD206 were not significantly affected, suggesting attenuation of M1-like pro-inflammatory signatures rather than a definitive shift toward an M2 phenotype in the spinal cord following rTMS treatment (**Figures 2A, B**).

We also examined MMPs, key inflammatory markers associated with microglial activity and implicated in NP pathophysiology through the release of inflammatory cytokines (61). Inhibition of these inflammatory markers represents a potential therapeutic strategy for NP. Specifically, MMP9 and MMP2 contribute to extracellular matrix degradation, playing a crucial role in NP induction and maintenance in peripheral nerve ligation models (62, 63). A previous report demonstrated that minocycline not only inhibited microglial activation but also reduced MMP9 levels after nerve injury, thereby suppressing neuroinflammation in NP (64, 65). Similarly, in our study, MMP9 expression was significantly reduced following rTMS treatment (**Figure 2C**), suggesting that rTMS exerts a comparable anti-inflammatory effect.

Additionally, we observed TRPV1 deactivation in the spinal cord and sciatic nerve following rTMS treatment (**Figures 2D** and **3A**), a molecule that plays a critical role in nociceptive synaptic modulation, as well as NP development and regulation (66, 67). Notably, TRPV1 activation has been directly linked to an increase in M1 microglia/macrophage markers and the production of inducible nitric oxide synthase, IL-6 and NLRP3 inflammasome, which are key mediators of pro-inflammatory responses (68, 69). Together, these findings support the interpretation that rTMS is associated with reduced glial-associated inflammatory responses in both the central and peripheral nervous systems, potentially contributing to NP attenuation.

In the sciatic nerve, Schwann cell markers S100 and GFAP were significantly elevated in NP rats (**Figure 3A**, **Supplementary Figure 2**). Schwann cells serve as the primary responders following nerve injury, and their interactions with macrophages play a crucial role in NP development and progression. Upon activation, both Schwann cells and macrophages release pro-inflammatory cytokines, including TNF-α, IL-1, and IL-6, which contribute to axonal damage and amplify nociceptor activity during the early stages of NP (11, 70). The reduction of these glial markers following rTMS treatment suggests attenuation of Schwann cell- and macrophage-associated inflammatory activity, consistent with the establishment of a less inflammatory microenvironment at the site of nerve injury.

Our NP model exhibited significant nerve degeneration and muscle atrophy (**Figures 4**, **5**), findings consistent with Wallerian degeneration, a nerve degeneration process that follows peripheral nerve injury (42, 43). Wallerian degeneration is characterized by axonal breakdown, myelin sheath loss, and subsequent muscle atrophy, all of which have been observed in NP rat models (71, 72). Against this pathological background, rTMS treatment was associated with improved myelin-related structural parameters in the injured sciatic nerve and attenuation of gastrocnemius muscle atrophy. Interestingly, several myelin-related parameters in the NP+rTMS group exceeded those observed in the control group, suggesting that the lower G-ratio and its distribution in **Figure 4C** may reflect active remyelination that had not yet reached a steady state at the time of analysis. Importantly, this pattern should not be interpreted as simple normalization toward the control state. Given that enhanced PI3K/Akt/mTOR signaling has been linked to excessive myelin growth and hypermyelination in myelinating glia, including Schwann cells, rTMS may also be associated with hypermyelination-like or otherwise aberrant remodeling (73, 74).

To explore potential mechanisms underlying rTMS-associated structural recovery, we examined the ERK and Akt signaling pathways. ERK and Akt are key signaling molecules that regulate essential cellular processes such as migration, differentiation, proliferation, and survival (23, 75). Additionally, ERK and Akt contribute to myelin sheath restoration by regulating Schwann cell de-differentiation and re-differentiation through the Nrg1/ErbB, ERK/MAPK, and PI3K/Akt/mTOR pathways (26, 76–79). Schwann cell de-differentiation facilitates myelin clearance via macrophage-mediated phagocytosis, while re-differentiation supports remyelination and nerve function recovery (41, 80, 81). In the present study, NP induction was associated with reduced ERK and Akt protein levels, whereas rTMS treatment was associated with restoration of these signaling proteins. However, because the phosphorylated-to-total ratios were not significantly altered, the present data do not permit a definitive conclusion regarding pathway-specific activation. Importantly, these findings do not demonstrate Schwann cell–specific signaling or cell fate changes. Rather, the observed changes may reflect recovery of overall ERK and Akt protein abundance, a broader improvement in the cellular environment, or both. Therefore, ERK- and Akt-related changes should be interpreted as indirect, tissue-level associations rather than evidence of Schwann cell–specific mechanisms. Taken together, these findings suggest that ERK- and Akt-related signaling changes are associated with the structural and inflammatory changes observed after rTMS treatment and may reflect a general improvement in cellular health, although they do not demonstrate that ERK/Akt signaling mediates the effects of rTMS.

Despite these findings, several limitations of this study should be considered. First, a sham-operated control group was not included. Although previous studies have reported minimal differences between sham-operated and naïve control animals in comparable NP models (14, 18), the potential contribution of surgical exposure and tissue manipulation cannot be completely excluded. Second, while the present results demonstrate associations among rTMS treatment, reduced inflammatory and glial markers, structural recovery of the sciatic nerve, and ERK/Akt-related signaling changes, the data do not establish direct mechanistic causality. Additional studies using pathway-specific or cell-specific approaches will be required to clarify the roles of these signaling pathways in rTMS-associated recovery. Third, a sham−rTMS group with matched anesthesia was not included. Previous studies in NP models have not been methodologically consistent, with some sham−rTMS−controlled experiments showing no significant difference between NP and NP + sham−rTMS groups (14, 18), whereas others omitted a sham−rTMS group (13, 53). In addition, isoflurane anesthesia itself may exert neuroprotective and anti-inflammatory effects, which could potentially confound the interpretation of rTMS-specific outcomes (82). Although outcome assessments were performed 24 hours later, potential cumulative or residual effects of repeated anesthesia cannot be fully excluded. Future studies incorporating an anesthesia−matched sham−rTMS control will be needed to better disentangle rTMS−specific effects from anesthetic confounding.

## 5 Conclusion

In conclusion, this study demonstrates that rTMS treatment was associated with modulation of neuroinflammatory and structural changes in both the spinal cord and sciatic nerve in a rat model of NP. rTMS was associated with reduced pro-inflammatory markers in the spinal cord, decreased glial markers in the sciatic nerve, improved myelin-related structural parameters of the injured sciatic nerve, and attenuation of muscle atrophy. These findings suggest that rTMS may influence NP through coordinated modulation of central and peripheral inflammatory processes. Furthermore, ERK- and Akt-related signaling may be associated with Schwann cell–related nerve recovery following rTMS.

## Data Availability

The original contributions presented in the study are included in the article/**Supplementary Material**. Further inquiries can be directed to the corresponding author.
