# Supplementary material for: Repetitive transcranial magnetic stimulation suppresses glia-associated neuroinflammation and promotes peripheral nerve recovery in neuropathic pain
Source: Front Immunol. 2026 May 7;17:1830638. doi: 10.3389/fimmu.2026.1830638 (PMC13189952; doi:10.3389/fimmu.2026.1830638)
Supplement: Supplementary file 1 [file DataSheet1.docx]

Supplementary Material

**Supplementary Table 1. Key resources used in the project.**

| **REAGENT/RESOURCE** | **SOURCE** | **IDENTIFIER** |
| --- | --- | --- |
| **Experimental models: Organisms/ strains** | | |
| Rat: Male Sprague–Dawley | SAMTAKO, Republic of Korea |  |
| **Oligonucleotides** | | |
| PCR Primer: CD40  Forward: TGCATGGGTGTCAGCCTGTTG  Reverse: GGCAGGCACGAAAGTTAGCTG | BIONEER, Republic of Korea | Gene ID: 171369 |
| PCR Primer: CD86  Forward: GTGAATGCCAAGTACCTGGGC  Reverse: GTTGGCGATCACTGAGAGCTC | BIONEER, Republic of Korea | Gene ID: 56822 |
| PCR Primer: Arg-1  Forward: CTCGAGGAGGGGTAGAGAAAG  Reverse: TACCACAGCAGCCAGCTGTTC | BIONEER, Republic of Korea | Gene ID: 29221 |
| PCR Primer: CD206  Forward: AGCACAGTCCATCCCTCAGTG  Reverse: CCCCAGACAGAAGAGTAGACC | BIONEER, Republic of Korea | Gene ID: 89808 |
| PCR Primer: MMP2  Forward: AGCTCCCGGAAAAGATTGAT  Reverse: GGCAGGCACGAAAGTTAGCTG | BIONEER, Republic of Korea | Gene ID: 81686 |
| PCR Primer: MMP9  Forward: TCGCTCGGATGGTTATCGC  Reverse: AAGACGCACATCTCTCCTGC | BIONEER, Republic of Korea | Gene ID: 81687 |
| PCR Primer: Glyceraldehyde-3-phosphate dehydrogenase  Forward: GGGCTGGCATTGCTCTCAATG  Reverse: AGGGAGATGCTCAGTGTTGGG | BIONEER, Republic of Korea | Gene ID: 24383 |
| **Software and algorithms** | | |
| GraphPad Prism 8.0 | GraphPad Software, USA | https://www.graphpad.com/features |
| Fiji (ImageJ 1.54f) | National Institutes of Health, USA | https://imagej.net/software/fiji/downloads |
| MyelTracer | [34] | https://github.com/HarrisonAllen/MyelTracer |
| **Others** | | |
| von Frey dynamic plantar aesthesiometer | Ugo Basile, Italy |  |
| Hot/Cold Plate | Ugo Basile, Italy |  |
| Eclipse Ts2 microscope | Nikon Instruments, Japan |  |
| Transmission electron microscopy (TEM) | Research Equipment Center, Korea Basic Science Institute., Republic of Korea |  |

**Supplementary Table 2. Descriptive statistics for thermal latency (hot plate).**

|  | **Thermal latency (s)** | | | **P-value** | |
| --- | --- | --- | --- | --- | --- |
|  | **Control**  **(N = 10)** | **NP**  **(N = 10)** | **NP+rTMS**  **(N = 9)** | **Comparisons among groups**  **at each time point** | **Comparisons of time points**  **within each group** |
| **Pre-NP**  **(Baseline)** | 20.40 ± 2.35 | 20.50 ± 2.65 | 18.78 ± 1.83 | Control *vs* NP  : p = 0.9996  Control *vs* NP+rTMS  : p = 0.850  NP *vs* NP+rTMS  : p = 0.856 | Pre-NP (Baseline) vs. D3 |
|  |  |  |  |  | Control: p = 0.991  NP: p = 0.210  NP+rTMS: p = 0.132 |
|  |  |  |  |  | Pre-NP (Baseline) vs. D5 |
|  |  |  |  |  | Control: p = 0.963  NP: p = 0.334  NP+rTMS: p = 0.974 |
| **D3** | 18.65 ± 2.47 | 13.72 ± 1.42 | 13.67 ± 0.91 | Control *vs* NP  : p = 0.229  Control *vs* NP+rTMS  : p = 0.185  NP *vs* NP+rTMS  : p = 0.999 | Pre-NP (Baseline) vs. D8 |
|  |  |  |  |  | Control: p > 0.9999  NP: p = 0.675  NP+rTMS: p = 0.982 |
|  |  |  |  |  | Pre-NP (Baseline) vs. D11 |
|  |  |  |  |  | Control: p = 0.915  NP: p = 0.235  NP+rTMS: p = 0.992 |
| **D5** | 17.95 ± 1.81 | 14.00 ± 1.63 | 17.50 ± 1.20 | Control *vs* NP  : p = 0.264  Control *vs* NP+rTMS  : p = 0.977  NP *vs* NP+rTMS  : p = 0.228 | D3 vs. D5 |
|  |  |  |  |  | Control: p = 0.992  NP: p > 0.997  NP+rTMS: p = 0.017 |
|  |  |  |  |  | D3 vs. D8 |
|  |  |  |  |  | Control: p = 0.949  NP: p = 0.961  NP+rTMS: p = 0.002 |
| **D8** | 20.00 ± 1.98 | 15.22 ± 2.25 | 19.94 ± 1.09 | Control *vs* NP  : p = 0.276  Control *vs* NP+rTMS  : p = 0.9997  NP *vs* NP+rTMS  : p = 0.187 | D3 vs. D11 |
|  |  |  |  |  | Control: p = 0.989  NP: p = 0.891  NP+rTMS: p = 0.106 |
|  |  |  |  |  | D5 vs. D8 |
|  |  |  |  |  | Control: p = 0.615  NP: p = 0.958  NP+rTMS: p = 0.231 |
| **D11** | 17.40 ± 1.83 | 11.83 ± 1.54 | 17.78 ± 0.92 | Control *vs* NP  : p = 0.079  Control *vs* NP+rTMS  : p = 0.981  NP *vs* NP+rTMS  : p = 0.014 | D5 vs. D11 |
|  |  |  |  |  | Control: p = 0.998  NP: p = 0.748  NP+rTMS: p = 0.9997 |
|  |  |  |  |  | D8 vs. D11 |
|  |  |  |  |  | Control: p = 0.281  NP: p = 0.355  NP+rTMS: p = 0.576 |

Data presented as mean ± SEM. Two-way repeated-measures ANOVA followed by Tukey’s post hoc multiple-comparisons test was used for statistical analysis. Mean/SEM values are rounded up to 2 decimal places, and p-values are rounded up to 3 or 4 decimal places.

**Supplementary Table 3. Descriptive statistics for mechanical withdrawal threshold (von Frey).**

|  | **Mechanical withdrawal force (g)** | | | **P-value** | |
| --- | --- | --- | --- | --- | --- |
|  | **Control**  **(N = 10)** | **NP**  **(N = 10)** | **NP+rTMS**  **(N = 9)** | **Comparisons among groups**  **at each time point** | **Comparisons of time points**  **within each group** |
| **Pre-NP**  **(Baseline)** | 7.81 ± 0.30 | 7.71 ± 0.37 | 7.77 ± 0.39 | Control *vs* NP  : p = 0.976  Control *vs* NP+rTMS  : p = 0.996  NP *vs* NP+rTMS  : p = 0.994 | Pre-NP (Baseline) vs. D3 |
|  |  |  |  |  | Control: p = 0.982  NP: p < 0.0001  NP+rTMS: p < 0.0001 |
|  |  |  |  |  | Pre-NP (Baseline) vs. D5 |
|  |  |  |  |  | Control: p = 0.047  NP: p < 0.0001  NP+rTMS: p = 0.0001 |
| **D3** | 7.69 ± 0.42 | 2.16 ± 0.08 | 2.09 ± 0.04 | Control *vs* NP  : p < 0.0001  Control *vs* NP+rTMS  : p < 0.0001  NP *vs* NP+rTMS  : p = 0.713 | Pre-NP (Baseline) vs. D8 |
|  |  |  |  |  | Control: p = 0.997  NP: p < 0.0001  NP+rTMS: p = 0.0002 |
|  |  |  |  |  | Pre-NP (Baseline) vs. D11 |
|  |  |  |  |  | Control: p = 0.962  NP: p < 0.0001  NP+rTMS: p = 0.0005 |
| **D5** | 8.28 ± 0.37 | 2.95 ± 0.35 | 3.45 ± 0.16 | Control *vs* NP  : p < 0.0001  Control *vs* NP+rTMS  : p < 0.0001  NP *vs* NP+rTMS  : p = 0.421 | D3 vs. D5 |
|  |  |  |  |  | Control: p = 0.325  NP: p = 0.224  NP+rTMS: p = 0.0001 |
|  |  |  |  |  | D3 vs. D8 |
|  |  |  |  |  | Control: p = 0.983  NP: p = 0.030  NP+rTMS: p = 0.0012 |
| **D8** | 7.92 ± 0.46 | 2.75 ± 0.18 | 3.64 ± 0.25 | Control *vs* NP  : p < 0.0001  Control *vs* NP+rTMS  : p < 0.0001  NP *vs* NP+rTMS  : p = 0.027 | D3 vs. D11 |
|  |  |  |  |  | Control: p = 0.948  NP: p = 0.098  NP+rTMS: p = 0.0019 |
|  |  |  |  |  | D5 vs. D8 |
|  |  |  |  |  | Control: p = 0.844  NP: p = 0.937  NP+rTMS: p = 0.975 |
| **D11** | 7.98 ± 0.41 | 2.78 ± 0.22 | 3.93 ± 0.25 | Control *vs* NP  : p < 0.0001  Control *vs* NP+rTMS  : p < 0.0001  NP *vs* NP+rTMS  : p = 0.030 | D5 vs. D11 |
|  |  |  |  |  | Control: p = 0.571  NP: p = 0.817  NP+rTMS: p = 0.875 |
|  |  |  |  |  | D8 vs. D11 |
|  |  |  |  |  | Control: p = 0.9997  NP: p = 0.9998  NP+rTMS: p = 0.934 |

Data presented as mean ± SEM. Two-way repeated-measures ANOVA followed by Tukey’s post hoc multiple-comparisons test was used for statistical analysis. Mean/SEM values are rounded up to 2 decimal places, and p-values are rounded up to 3 or 4 decimal places.


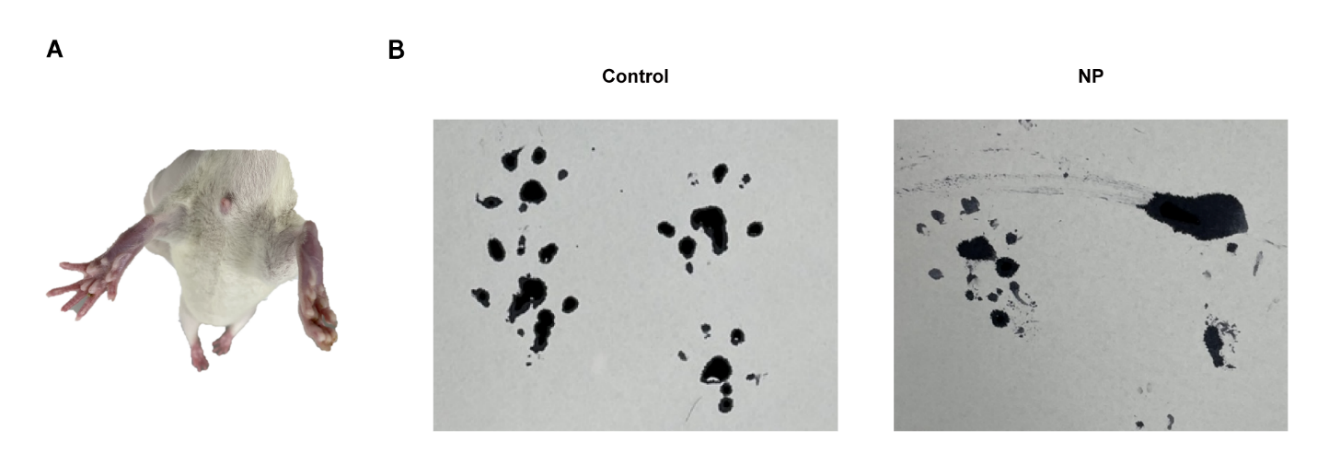


**Supplementary Figure 1. Disturbed gait after NP induction.** (A) After the surgery, the right side of the foot shape was changed. (B) The difference of footprints between the control and the NP. The paws of the rats were stained with an ink mixture (alcohol and glycerin) and allowed to walk on a piece of white paper. The NP procedure leads to less distinct prints of the heel paw affected by the NP procedure and a disturbed gait (dragging of the affected leg), due to guarding of the painful paw, compared to the unaffected paw on the contralateral side of the body.


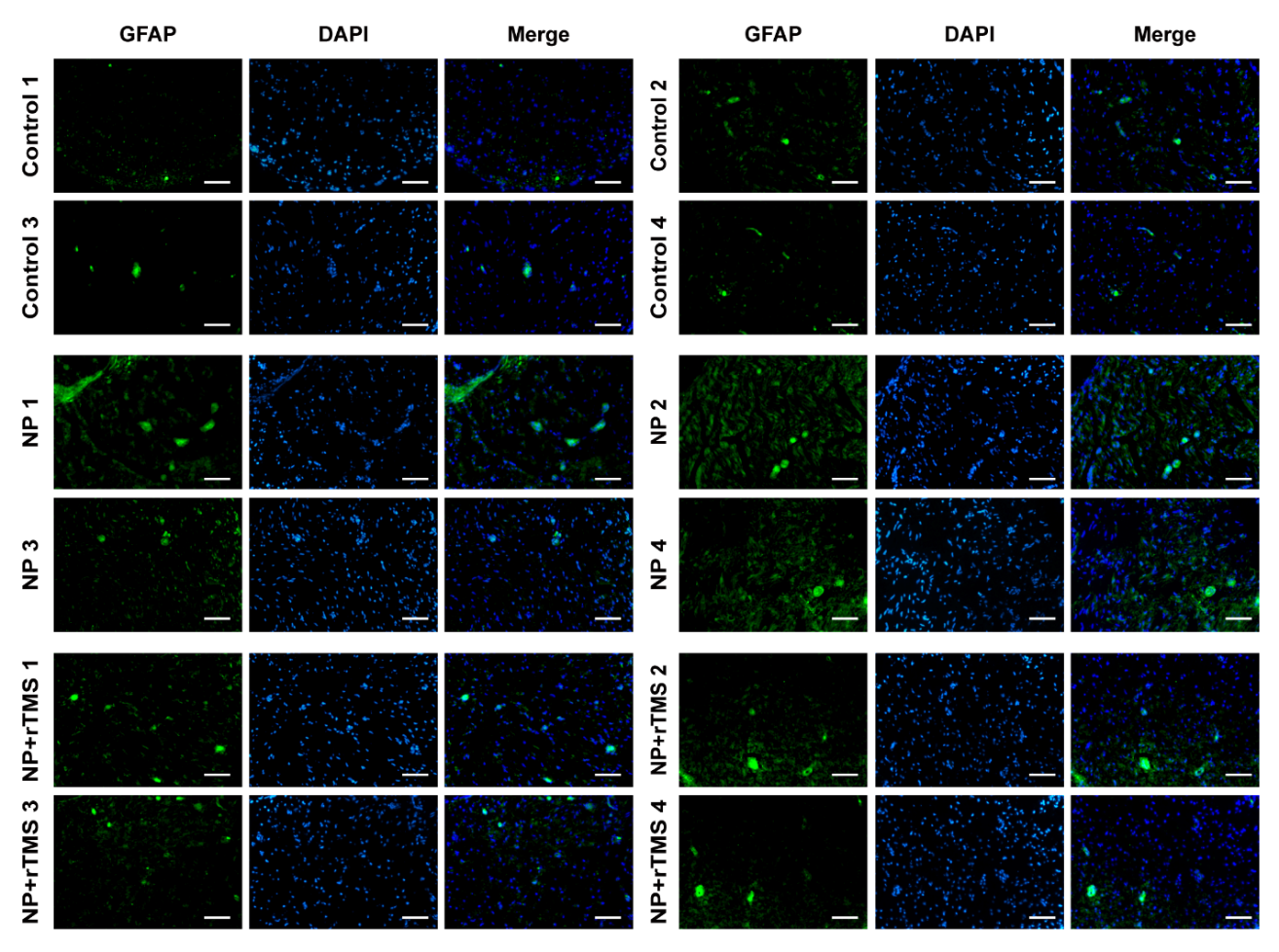


**Supplementary Figure 2. Immunofluorescence images of GFAP in the sciatic nerve.** Representative immunofluorescence images for GFAP (green), DAPI (blue), and merged in the sciatic nerve of Control, NP, and NP+rTMS mice. Images represent one sample each group (n=4 per group). Scale bar: 50 μm.

**
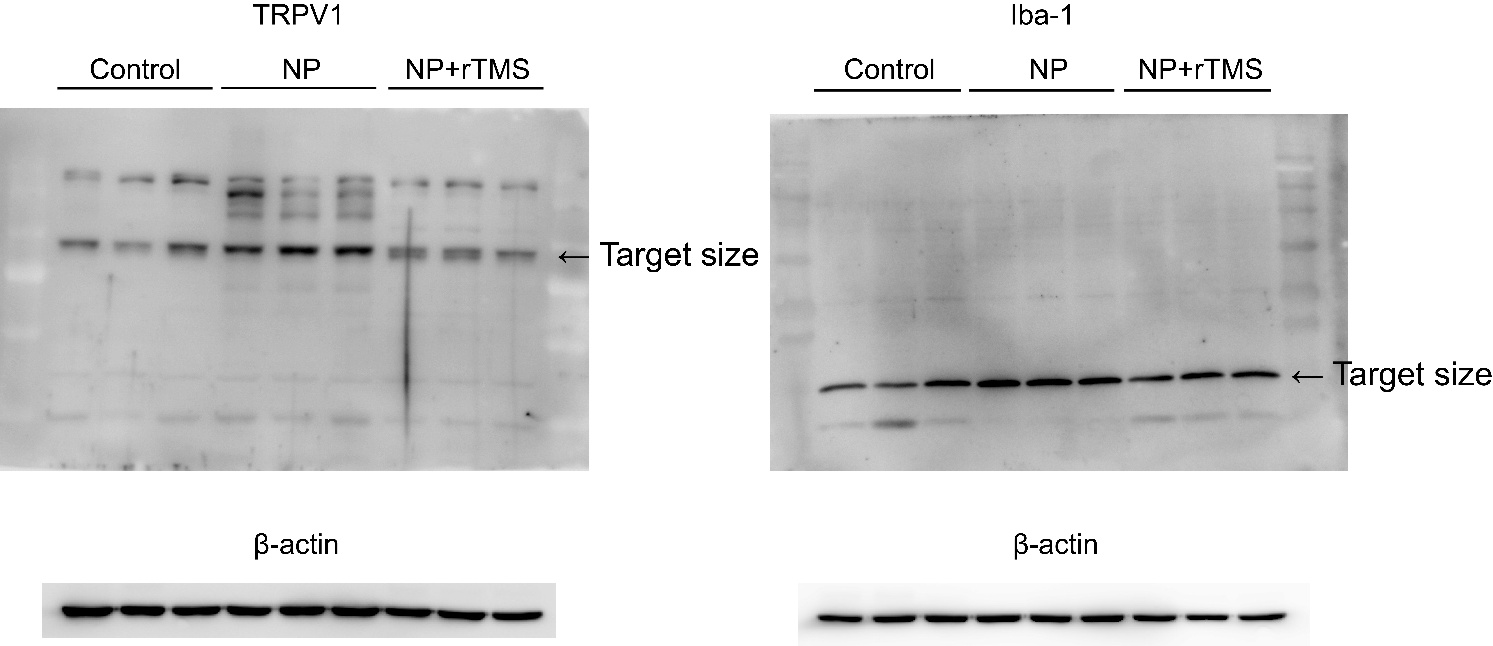
**

**Supplementary Figure 3. Original images of PVDF blots presented in Fig. 2D.** The samples are loaded as three consecutive lanes for each group (Control, NP, and NP+rTMS). The β-actin blots obtained from the same blot as and used for the quantification of the target proteins (TRPV1 and Iba-1) are presented at the bottom of the blots for its respective target protein.


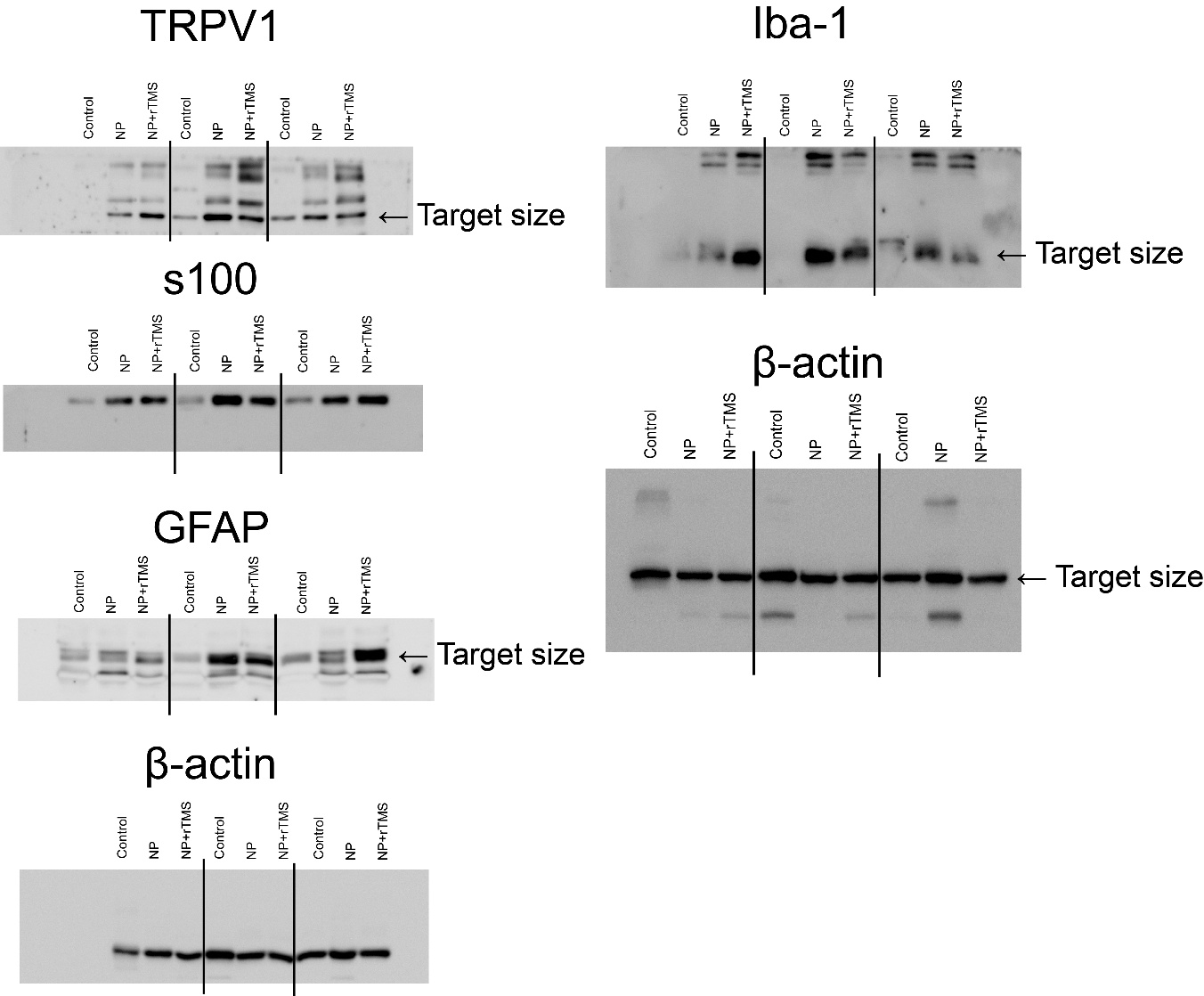


**Supplementary Figure 4. Original gel images from Western blots for Fig. 3A.** The samples are loaded in sets of three (Control, NP, NP+rTMS). A single blot is used for the detection of TRPV1, S100, and β-actin, followed by stripping to detect GFAP. Another single blot is used for the detection of Iba-1 and β-actin. Based on the predicted size of target proteins, the blot was cut at multiple sites to allow for the blotting of specific target protein.

**
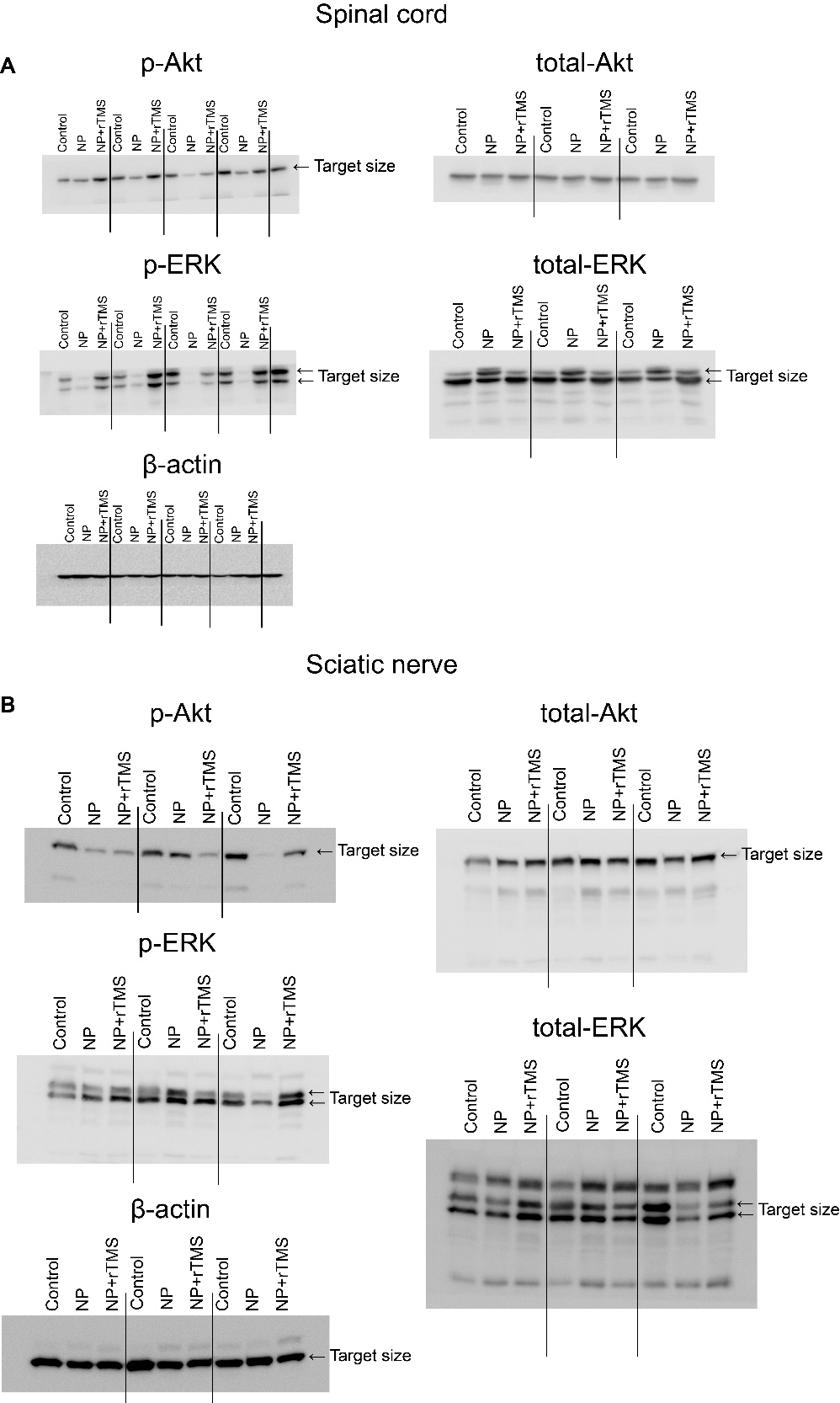
**

**Supplementary Figure 5. Original images of PVDF blots presented in Fig. 6. (A)** The spinal cord and (B) sciatic nerve samples are loaded in sets of three (Control, NP, NP+rTMS). A single blot is used for the detection of p-Akt, p-ERK, and β-actin, and another blot is used for the detection of total-Akt and total-ERK. Based on the predicted size of target proteins, the blot was cut at multiple sites to allow for the blotting of specific target proteins. Due to the overlap in size between p-ERK/total-ERK and β-actin, detection of β-actin is not possible on the same blot. A new gel is run with the same samples, to obtain solely the β-actin for quantification.
